# Extracellular Vesicles Derived From Human Umbilical Cord Mesenchymal Stem Cells Protect Against DOX-Induced Heart Failure Through the miR-100-5p/NOX4 Pathway

**DOI:** 10.3389/fbioe.2021.703241

**Published:** 2021-08-25

**Authors:** Zhenglong Zhong, Yuqing Tian, Xiaoming Luo, Jianjie Zou, Lin Wu, Julong Tian

**Affiliations:** Department of Cardiology, Affiliated Hospital of Panzhihua University, Panzhihua, China

**Keywords:** oxidative stress, miR-100-5p, NOX4, heart failure, human umbilical cord mesenchymal stem cells, extracellular vesicles

## Abstract

The end result of a variety of cardiovascular diseases is heart failure. Heart failure patients’ morbidity and mortality rates are increasing year after year. Extracellular vesicles (EVs) derived from human umbilical cord mesenchymal stem cells (HucMSC-EVs) have recently been discovered to be an alternative treatment for heart failure, according to recent research. In this study, we aimed to explore the underlying mechanisms in which HucMSC-EVs inhibited doxorubicin (DOX)-induced heart failure in AC16 cells. An miR-100-5p inhibitor and an miR-100-5p mimic were used to transfect HucMSCs using Lipofectamine 2000. HucMSC-EVs were isolated and purified using the ultracentrifugation method. AC16 cells were treated with DOX combined with HucMSC-EVs or an EV miR-100-5-p inhibitor or EV miR-100-5-p mimic. ROS levels were measured by a flow cytometer. The levels of LDH, SOD, and MDA were measured by biochemical methods. Apoptotic cells were assessed by a flow cytometer. Cleaved-caspase-3 and NOX4 protein expression were determined by Western blot. The experiment results showed that HucMSC-EVs inhibited DOX-induced increased levels of ROS, LDH, and MDA, and decreased levels of SOD which were reversed by an EV miR-100-5-p inhibitor, while EV miR-100-5-p mimic had a similar effect to HucMSC-EVs. At the same time, HucMSC-EV-inhibited DOX induced the increases of apoptotic cells as well as NOX4 and cleaved-caspase-3 protein expression, which were reversed by an EV miR-100-5-p inhibitor. Furthermore, the NOX4 expression was negatively regulated by miR-100-5p. Overexpression of NOX4 abolished the effects in which HucMSC-EVs inhibited DOX-induced ROS, oxidative stress, and apoptosis increases. In conclusion, these results indicate that HucMSC-EVs inhibit DOX-induced heart failure through the miR-100-5p/NOX4 pathway.

## Introduction

Heart failure is the final result of various cardiovascular diseases, among which the most common causes include coronary heart disease, hypertension, cardiomyopathy, and valvular heart disease. Its morbidity and mortality are gradually increasing year by year, posing a serious threat to human health. Mesenchymal stem cells (MSCs) are a type of pluripotent stem cells with multiple differentiation potentials that implant in the mesoderm (Jaquet et al., 2005). MSCs can be derived from the bone marrow, placenta, adipose tissue, umbilical cord blood, *etc*. Human umbilical cord mesenchymal stem cells (HucMSCs) are easier to obtain and have proliferation and immunosuppressive effects. In addition, there are no ethical issues for HucMSCs in clinical applications (Li et al., 2018; Guan et al., 2019; Xie et al., 2020). HucMSCs are used to increase the number of cardiomyocytes with contractile function, thereby improving heart failure caused by various reasons (Bartolucci et al., 2017; Mao et al., 2017; Kobayashi et al., 2018; Matsushita, 2020).

Paracrine function is the main way for mesenchymal stem cells to exert therapeutic effects. As one of the main components of paracrine, EVs include mRNA, miRNA, circRNA, protein, and other functional molecules, which not only participate in the regulation of cell proliferation and survival but also play an important role in signal transmission and cell communication (Keerthikumar et al., 2016; Tkach and Théry, 2016). EVs are formed by eukaryotic cells through endocytosis of some signal molecules to form multivesicular endosomes, and then fused with the cell membrane and released into nanovesicles in the extracellular environment. EVs contain different kinds of lipids, proteins, RNAs, and other biologically active molecules, which play a variety of biological functions. EVs have been proven to be an important medium for MSCs to exert therapeutic effects. They can enter the cytoplasm through endocytosis or direct fusion with the cell membrane, or through receptor–ligand interactions, to transmit biological information to target cells, thereby exerting biological functions and regulating abnormal microenvironment (Davidson and Yellon, 2018; Balbi and Vassalli, 2020). Recent studies have shown that MSC-derived EVs (MSC-EVs) are expected to become a new alternative to stem cell therapy for heart failure (Fang et al., 2016).

DOX is an antitumor drug widely used clinically. It has a good effect on many tumors. However, DOX has very serious cardiotoxicity and finally leads to heart failure. DOX-induced cardiotoxicity can be used as an *in vitro* model to summarize the mechanism of heart failure. The application of the *in vitro* cardiotoxicity test system can greatly help understand the development of heart failure (Sachinidis, 2020). In this study, the effects of HucMSC-EVs on DOX-induced heart failure were studied. The aim of this study was to explore the molecular mechanism of HucMSC-EVs inhibiting DOX-induced heart failure.

## Materials and Methods

### Cell Culture

Human AC16 cells were obtained from ATCC (Manassas, VA, United States) and cultured in a DMEM medium (Hyclone, SH30243.01, Logan, UT, United States) supplemented with 10% fetal bovine serum (Gibco, 16000e044, Carlsbad, CA, United States) and 1% penicillin–streptomycin (Solarbio, P1400, Beijing, China) and incubated at 37°C with 5% CO_2_. AC16 cells were treated with 2 µmol/L DOX (Sigma-Aldrich, 25316-40-9, Shanghai, China) and HucMSC-EVs in different concentrations for 24 h.

### Human Umbilical Cord Mesenchymal Stem Cell Characterization

HucMSCs were provided by Stem Cell Bank, Chinese Academy of Sciences and cultured in serum-free MSC NutriStem® XF Medium (Biological Industries, Beit HaEmek, Israel). When the cells grew to 80–90% confluence, they were digested with 0.25% trypsin containing 0.01% EDTA. The cells were resuspended in PBS and adjusted to 1 × 10^6^ cells/ml. The mouse antihuman CD34, CD45, CD44, and CD105 were added and incubated at 4°C in dark for 15–30 min. Surface antigens of MSCs were characterized by using a Beckman CytoFLEX flow cytometer (Beckman Coulter Life Sciences, Tokyo, Japan) and the Human MSC Analysis Kit (BD Biosciences, San Jose, CA, United States).

### Isolation and Characterization of Extracellular Vesicles

HucMSCs were cultured in EV-free production medium for 24–48 h before conditioned medium was collected. EVs were isolated and purified by ultracentrifugation according to the protocol (Théry et al., 2006). The morphology of isolated HucMSC-EVs was observed by transmission electron microscopy (FEI Tecnai G2 Spirit Twin, Philips, NL). The TSG101 and CD81 protein markers of EVs were detected by Western blot.

### Extracellular Vesicle Uptake Assay

PKH67 EV green fluorescent dye (UR52303, Umibio, China) was used to trace EVs being endocytosed by AC16 cells. In brief, EVs were stained with PKH67 dying working solution, in which the PKH liker was mixed with diluent C at a ratio of 1:9 in dark at room temperature. Then they were mixed well and incubated for 10 min in dark. The labeled EVs were incubated on AC16 cells for 24 h at 37°C, and cells were washed with 1 × PBS. Cells were mounted in a mounting medium containing DAPI (4′,6-diamidino-2-phenylindole dihydrochloride) (DAPI-Fluoromount-GTM, Yeasen Biotechnology, Shanghai, China). A laser-scanning confocal microscope was applied to take all of the images (Youn et al., 2019).

### Cell Transfection

The coding region of NOX4 (NM_001143837.2) was cloned into pCDNA3.1 (+) plasmids (Clontech, Mountain View, CA, United States) at Hind III and EcoR1 sites. It was designated to oeNOX4. oeNOX4 was transfected to AC16 cells using Lipofectamine 2000 Kit (Invitrogen, Carlsbad, CA, United States). The negative control included cells with blank vector pCDNA3.1 (+) transfection. The primers used for amplification of the coding sequence of NOX4 were as follows:

NOX4-F:5′-CCCAAGCTTATGAATGTCCTGCTTTTCTGGAAAAC-3' (Hind III)

NOX4-R: 5′-CGG​AAT​TCT​CAG​CTG​AAA​GAC​TCT​TTA​TTG​TAT​TC-3' (EcoR I)

### miRNA Transfection

miR-100-5p mimic (5′-AAC​CCG​UAG​AUC​CGA​ACU​UGU​G-3′), miR-100-5p inhibitor (5′-CAC​AAG​UUC​GGA​UCU​ACG​GGU​U-3′), and negative control (NC, 5′-CAG​UAC​UUU​UGU​GUA​GUA​CAA-3′) were synthesized by Beyotime (Beijing, China) and transfected to cells with Lipofectamine 2000 Kit individually.

### Luciferase Reporter Assay

The NOX4 reporter gene plasmid is constructed by gene synthesis. The NOX4 (NM_001143837.2) sequence was found in NCBI. According to the NOX4 3′-UTR sequence, wild-type and mutant NOX4 3′-UTR sequences with Sac I and Xho I sticky ends were synthesized. The mutation site was based on the binding site of hsa-miR-100-5p and NOX4 3′-UTR sequence. The NOX4 3′-UTR binding site sequence containing mutation was inserted into the vector pGL3-promoter through Sac I and Xho I restriction sites to construct pGL3-Promoter-mutNOX4 3′-UTR. Wild-type NOX4 3′-UTR was inserted into the vector pGL3-Promoter through Sac I and Xho I restriction sites to construct pGL3-Promoter-wtNOX4-3′-UTR. PGL3-Promoter vector had firefly fluorescent gene (luc2) and pRL-TK with Renilla fluorescent gene (hRluc). A map of the predicted binding site of hsa-miR-100-5p to NOX4 and mutant was as following.

**Table:** 

hsa-miR-100-5p	5′ GCCUAGAUGCCCAA 3′
—	| | | | | | |
wtNOX4-3′-UTR	5′ TATTGATACGGGTACT 3′
mutNOX4-3′-UTR	5′ TATTGAGCATTAGCCT 3

293T cells were then co-transfected with miR-100-5p inhibitor, miR-100-5p mimic and pGL3-NOX4-WT or miR-100-5p inhibitor, miR-100-5p mimic, and pGL3-NOX4-MUT plasmids. After transfection, the cells were treated with a Dual-Luciferase Reporter Gene Detection System Test Kit. The firefly luciferase activity and Renilla luciferase activity were detected by a microplate reader. Luciferase activity ratio in this study was the ratio of the firefly luciferase activity to Renilla luciferase activity.

### ROS Detection

A dichlorodihydrofluorescein diacetate (DCFH-DA) fluorescent probe (Sigma-Aldrich, D6883, Shanghai, China) combined with the flow cytometric analysis was used to detect the changes of reactive oxygen species (ROS) levels. The reactive oxygen species in the cell can oxidize nonfluorescent DCFH to produce fluorescent DCF. By detecting the fluorescence of DCF, the level of reactive oxygen species in the cell can be known. According to the production of red fluorescence in living cells, the amount and change of the cell ROS content can be judged. Briefly, AC16 cells were resuspended in 1x PBS, and the density was adjusted to 5 × 10^5^ cells/ml. AC16 cells were then incubated with 10 μM DCFH-DA for 20 min in dark at 37°C and subsequently subjected to the flow cytometric analysis (Shi et al., 2016).

### Cell Apoptosis

AC16 cells were seeded in 6-well plates with 1 × 10^5^ per well and cultured for 12–24 h before use. AC16 cells were harvested 24 h after being treated with DOX combined with HucMSC-EVs or EV miR-100-5-p inhibitor or oeNOX4. Cells were prepared with the Annexin V-FITC Apoptosis Detection Kit (Beyotime, C1062s, Beijing, China) according to the manufacturer’s recommendations. Briefly, AC16 cells were centrifuged at 1,000 g for 5 min and resuspended to a concentration of 1 × 10^6^ cells/ml. 1 × 10^6^ resuspended cells were centrifuged at 1,000 g for 5 min. The supernatant was discarded, and 195 μL of Annexin V-FITC binding solution was added to gently resuspend the cells. And 10 μL of Annexin V-FITC was added and mixed gently. Then 5 μL of propidium iodide staining solution was added to the mix gently. The cell suspension was gently vortexed and incubated in dark at room temperature for 15 min, and then placed in an ice bath. At the same time, a tube without Annexin V-FITC and PI was used as a negative control. Flow cytometry was performed within 1 h. The following method was used: The Annexin V-negative/PI-negative part represented viable cells. The Annexin V-positive/PI-negative part represented early apoptotic cells, and the Annexin V-positive/PI-positive part represented late apoptotic and dead cells.

### Biochemical Detection

The levels of lactate dehydrogenase (LDH), superoxide dismutase (SOD), and malondialdehyde (MDA) in cells were measured, respectively, using the LDH (A020-2), SOD (A001-3), and MDA (A003-1) kits (Jiancheng Biotechnology Research Institute, Nanjing, Jiangsu, China) according to the manufacturer's recommendations. Assays were performed in triplicate, and the mean values of each sample were calculated manually.

#### QRT-PCR

Total RNA was extracted using Trizol reagent (Invitrogen, Carlsbad, CA, United States) according to the manufacturer’s protocol, and reverse-transcribed with a RevertAid First Strand cDNA Synthesis Kit (Thermo Fisher Scientific Inc., Waltham, MA, United States). qRT-PCR was done using an SYBR green PCR Master Mix (Thermo Fisher Scientific Inc., Waltham, MA, United States) on the ABI 7300 system. The relative abundance of genes was quantified by using the comparative 2-ΔΔCt with β-actin or U6 as an internal control. The sequences of primers used in the study were as follows. Human NOX4, Primer F:

5′-TTT​AGA​TAC​CCA​CCC​TCC​CG-3′, Primer R:

5′-GGC​ACA​GTA​CAG​GCA​CAA​AGG-3′. Human cytochrome b-245 beta chain (CYBB), Primer F: 5′-CTA​AGA​TAG​CGG​TTG​ATG​GGC-3′, Primer R:

5′-CTT​GAG​AAT​GGA​TGC​GAA​GG-3′. Human β-actin, Primer F:

5′-CGT​GGA​CAT​CCG​CAA​AGA​C-3′, Primer R: 5′-TGC​TGG​GAG​CCA​GAG​CAG-3'.

hsa-miR-100-5p, RT Primer:

5′-GTC​GTA​TCC​AGT​GCA​GGG​TCC​GAG​GTA​TTC​GCA​CTG​GAT​ACG​ACC​ACA​A

G-3′, Primer F: 5′-GCG​AAC​CCG​TAG​ATC​CGA​A-3′, Primer R:

5′-AGT​GCA​GGG​TCC​GAG​GTA​TT-3′. Human U6, Primer F:

5′-CTC​GCT​TCG​GCA​GCA​CA, Primer R: 5′-AAC​GCT​TCA​CGA​ATT​TGC​GT-3′.

### Western Blot

AC16 cells were lysed by the addition of RIPA lysis buffer supplemented with a protease and phosphatase inhibitor cocktail (p8340 and p8250, Sigma, St Louis, MO, United States). 25 µg of total protein was separated by SDS-PAGE and transferred onto a nitrocellulose membrane. Membranes were further blocked with 5% skim milk and immersed into antibody solutions against TSG101 (1:2000, ab120511, Abcam, Cambridge, MA, United States), CD81 (1:2000, ab109201, Abcam, Cambridge, MA, United States), NOX2 (1:5000, ab129068, Abcam, Cambridge, MA, United States), cleaved-caspase-3 (1:500, ab13847, Abcam, Cambridge, MA, United States), β-actin (1:2000, ab8226, Abcam, Cambridge, MA, United States), and NOX4 (1:2000, 14347-1-AP, Proteintech, Rosemont, IL, United States). Then the membranes were immersed into the secondary antibody solution linked to horseradish peroxidase (A0208 and A0216, Beyotime, Shanghai, China). Signals were captured by a chemiluminescence system.

### Statistical Analysis

All data were expressed as mean ± standard deviation (SD). One-way analysis of variance (ANOVA) was applied to assess the statistically significant differences between more than two groups. Statistical analysis was performed by Prism 8.0.2 software (GraphPad, San Diego, United States). *p* value < 0.05 was considered significant.

## Results

### Identification of Human Umbilical Cord Mesenchymal Stem Cell-Extracellular Vesicles

**FIGURE 1:**
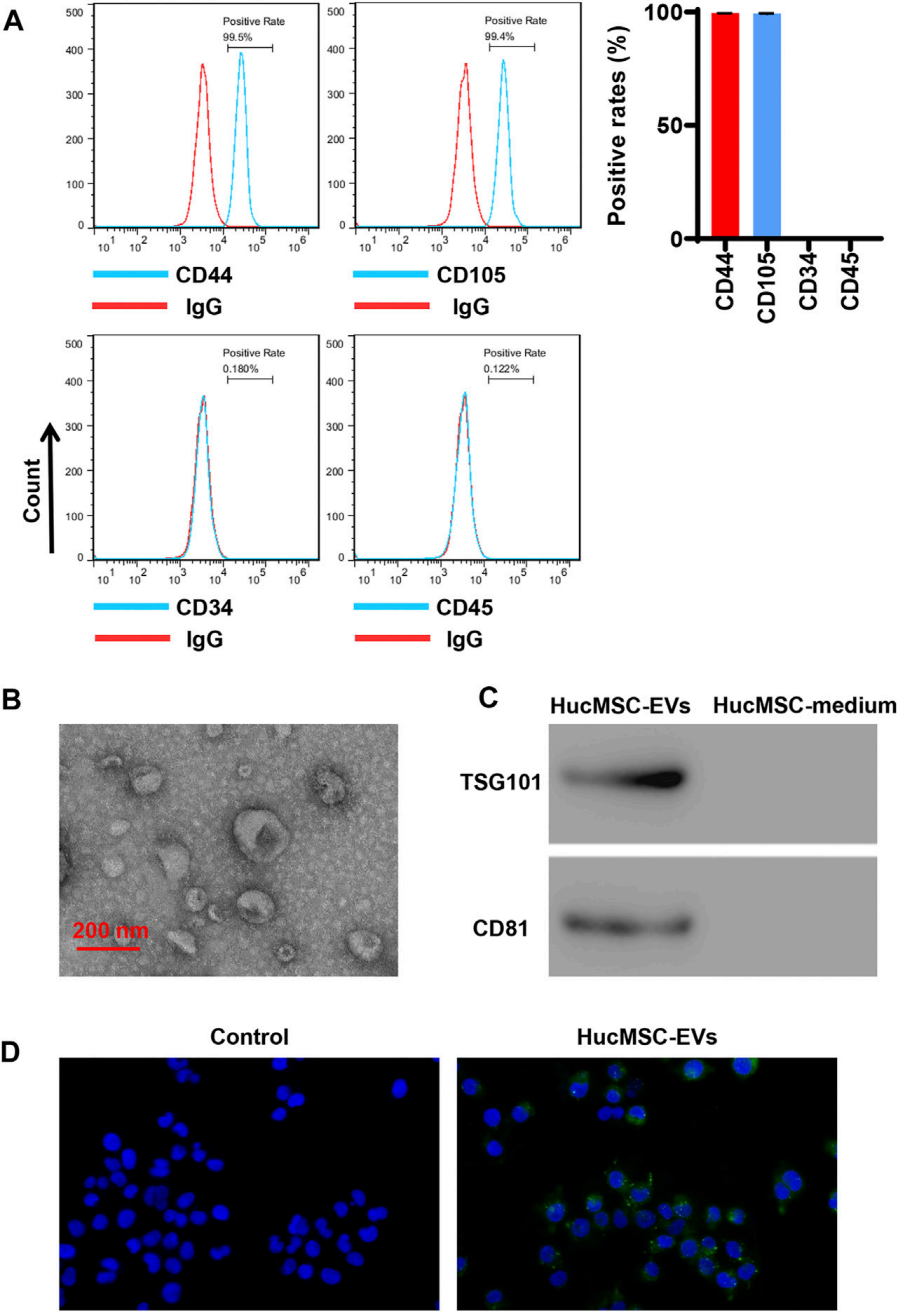
Isolation and identification of HucMSC-EVs. **(A)** The surface markers CD44 and CD105 of HucMSCs were identified by flow cytometry. **(B)** The morphology of purified HucMSC-EVs was observed by transmission electron microscopy: scale bar: 200 nm. **(C)** The markers CD81 and TSG101 of HucMSC-EVs were detected by Western blot. **(D)** HucMSC-EV endocytosis traced by PKH67 was observed by a laser scanning microscope in AC16 cells.

HucMSCs were cultured and collected. HucMSCs were identified by a flow cytometer to detect their surface markers. Flow cytometry exhibited that HucMSC surface markers, such as CD44 and CD105, were highly expressed, while hematopoietic stem marker CD34 and leukocyte surface antigen CD45 exhibited low expression (Figure 1A). Next, HucMSC-EVs were extracted and identified. Transmission electron microscope showed that HucMSC-EVs were small round or elliptical membranous bi-lipid membrane vesicles. Their diameters ranged in size from 30 to 100 nm. There were low electron densities in the vesicles (Figure 1B). The specific markers, namely, TSG101 and CD81, of HucMSC-EVs were detected by Western blot. While there were no expression for the specific markers TSG101 and CD81 of HucMSC-EVs in the HucMSC medium (Figure 1C). HucMSC-EVs were labeled by PKH67 and incubated with AC16 cells for 24 h. Under a laser scanning microscope, the green EVs were located in the cytoplasm (Figure 1D). These results suggest that HucMSC-EVs were successfully isolated.

### Human Umbilical Cord Mesenchymal Stem Cell-Extracellular Vesicles Inhibit Doxorubicin-Induced Oxidative Stress and Apoptosis

**FIGURE 2:**
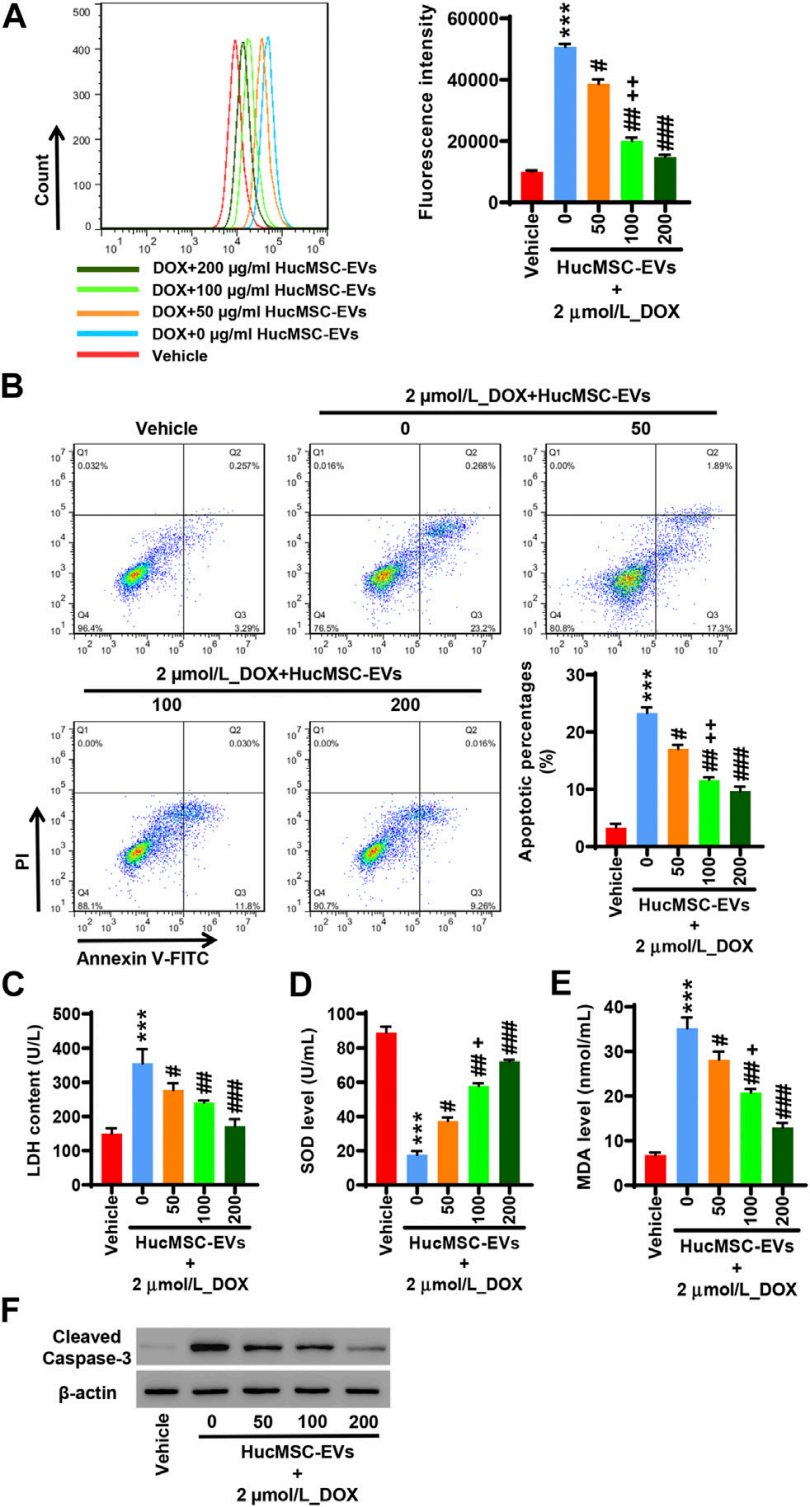
HucMSC-EVs inhibit DOX-induced oxidative stress and apoptosis. AC16 cells were treated with 2 µmol/L DOX and HucMSC-EVs at different concentrations (0, 50, 100, and 200 μg/ml) for 24 h. **(A)** ROS levels were measured by flow cytometry. **(B)** The percentages of apoptotic cells were assessed by flow cytometry. Quadrant 1 represented dead cells. Quadrant 2 represented late apoptotic cells. Quadrant 3 represented early apoptotic cells. Quadrant 4 represented normal cells. **(C–E)** The levels of LDH **(C)**, SOD **(D)**, and MDA **(E)** were measured with biochemistry methods. **(F)** Western blot analysis of cleaved-caspase-3. ^***^ *p* < 0.001 vs. vehicle, ^#^ *p* < 0.05, ^##^ *p* < 0.01, ^###^ *p* < 0.001 vs. 2 μmol/L_DOX + 0 μg/ml_HucMSC-EVs, ^+^ *p* < 0.05, ^++^ *p* < 0.01 vs. 2 μmol/L_DOX + 50 μg/ml_HucMSC-EVs.

AC16 cells were treated with 2 µmol/L DOX and HucMSC at concentrations of 0 μg/ml, 50 μg/ml, 100 μg/ml, and 200 μg/ml for 24 h. Flow cytometry showed that 2 µmol/L DOX obviously induced ROS levels to increase, while HucMSC-EV treatment decreased ROS levels that were increased by DOX. HucMSC-EVs significantly reduced ROS levels at the concentrations of 50 μg/ml, 100 μg/ml, and 200 μg/ml. The effect of HucMSC-EV treatment was concentration dependent (Figure 2A). Meanwhile, 2 µmol/L DOX increased LDH release and MDA levels, and decreased SOD levels. However, HucMSC-EV treatment inhibited the increases of LDH release and MDA levels, and the decreases of SOD levels which were induced by DOX. The functions of HucMSC-EVs on LDH release, SOD levels, and MDA levels were in a concentration-dependent manner (Figures 2C–E). Furthermore, 2 µmol/L DOX induced AC16 cell apoptosis. Flow cytometry displayed that HucMSC-EVs reduced apoptotic cells that were increased by DOX with the concentrations of 50, 100, and 200 μg/ml. The action of HucMSC-EVs on AC16 cell apoptosis was in a concentration-dependent manner (Figure 2B). Western blot exhibited that HucMSC-EVs markably repressed the cleaved-caspase-3 expression with the different concentrations of 50, 100, and 200 μg/ml, which were highly expressed by DOX (Figure 2F). These findings suggest that HucMSC-EVs obviously inhibit cardiomyocyte oxidative stress and apoptosis that are induced by DOX in AC16 cells.

### Human Umbilical Cord Mesenchymal Stem Cell-Extracellular Vesicles Inhibit NOX4 Expression Induced by Doxorubicin

**FIGURE 3:**
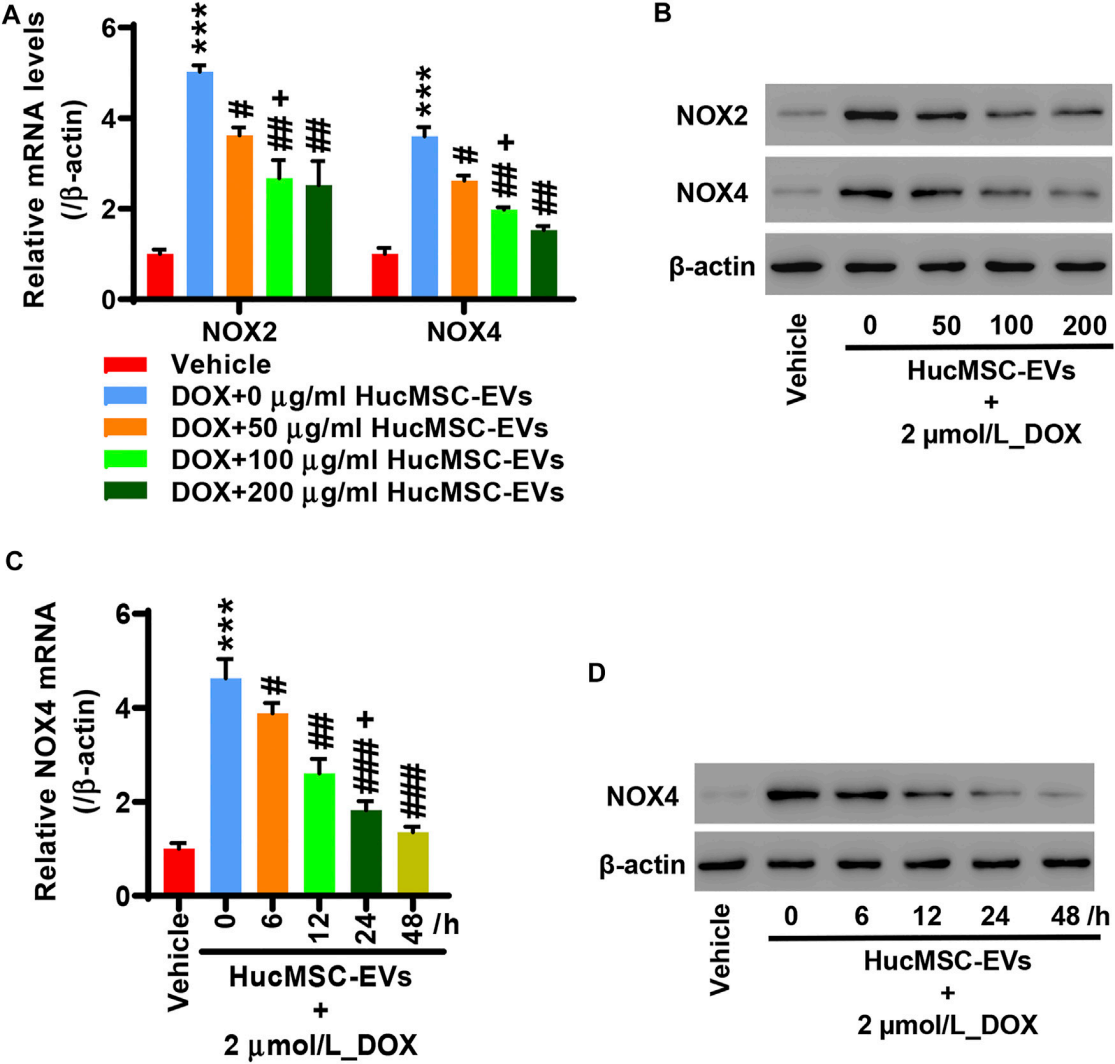
HucMSC-EVs inhibits NOX4 expression. AC16 cells were treated with 2 µmol/L DOX and HucMSC-EVs at different concentrations (0, 50, 100, and 200 μg/ml) for 24 h. **(A)** qRT-PCR and **(B)** Western blot were performed to detect NOX2 and NOX4 expression, respectively. ^***^ *p* < 0.001 vs. vehicle, ^#^ *p* < 0.05, ^##^ *p* < 0.01 vs. 2 μmol/L_DOX + 0 μg/ml_HucMSC-EVs, ^+^ *p* < 0.05 vs. 2 μmol/L_DOX + 50 μg/ml_HucMSC-EVs. **(C–D)** AC16 cells were treated with 2 µmol/L DOX and HucMSC-EVs at a concentration of 100 μg/ml for different times (0, 6, 12, 24, and 48 h). **(C)** qRT-PCR and **(D)** Western blot were used for the detection of NOX4 expression. ^***^ *p* < 0.001 vs. Vehicle, ^#^ *p* < 0.05, ^##^ *p* < 0.01, ^###^ *p* < 0.001 vs. 0 h, ^+^ *p* < 0.05 vs. 12 h.

AC16 cells were treated with 2 µmol/L DOX and HucMSC-EVs with the concentrations of 0, 50, 100, and 200 μg/ml for 24 h. qRT-PCR indicated that DOX extremely increased the NOX2 and NOX4 mRNA expression. HucMSC-EVs could repress the mRNA expression of NOX2 and NOX4 at concentrations of 50, 100, and 200 μg/ml, which were increased by DOX. Compared to the 2 μmol/L_DOX+50 μg/ml_HucMSC-EV group, HucMSC-EV treatment at 100 μg/ml apparently suppressed NOX2 and NOX4 mRNA expression, which was induced by DOX (Figure 3A). Western blot displayed that HucMSC-EVs suppressed protein expression of NOX2 and NOX4 with the concentrations of 50, 100, and 200 μg/ml, which was increased by DOX (Figure 3B). AC16 cells were treated with 2 µmol/L DOX and 100 μg/ml HucMSC-EVs for 0, 6, 12, 24, and 48 h. qRT-PCR showed that 2 µmol/L DOX remarkably induced NOX4 mRNA expression increase. HucMSC-EVs attenuated NOX4 mRNA expression with a concentration of 100 μg/ml at 6, 12, 24, and 48 h, which were increased by DOX. Compared to 12 h, HucMSC-EV treatment markedly suppressed NOX4 mRNA expression with 100 μg/ml at 24 h, which was increased by DOX (Figure 3C). Similarly, Western blot displayed that the NOX4 protein expression was ameliorated at a concentration of 100 μg/ml for 6, 12, 24, and 48 h, which was increased by DOX (Figure 3D). Collectively, the data suggest that HucMSC-EVs markedly inhibit NOX4 expression in a time- and concentration-dependent manner, which is induced by DOX.

### Inhibition of EV miR-100-5-p Reverses Those Effects That Human Umbilical Cord Mesenchymal Stem Cell-Extracellular Vesicles Inhibit Doxorubicin-Induced Oxidative Stress and Apoptosis

**FIGURE 4:**
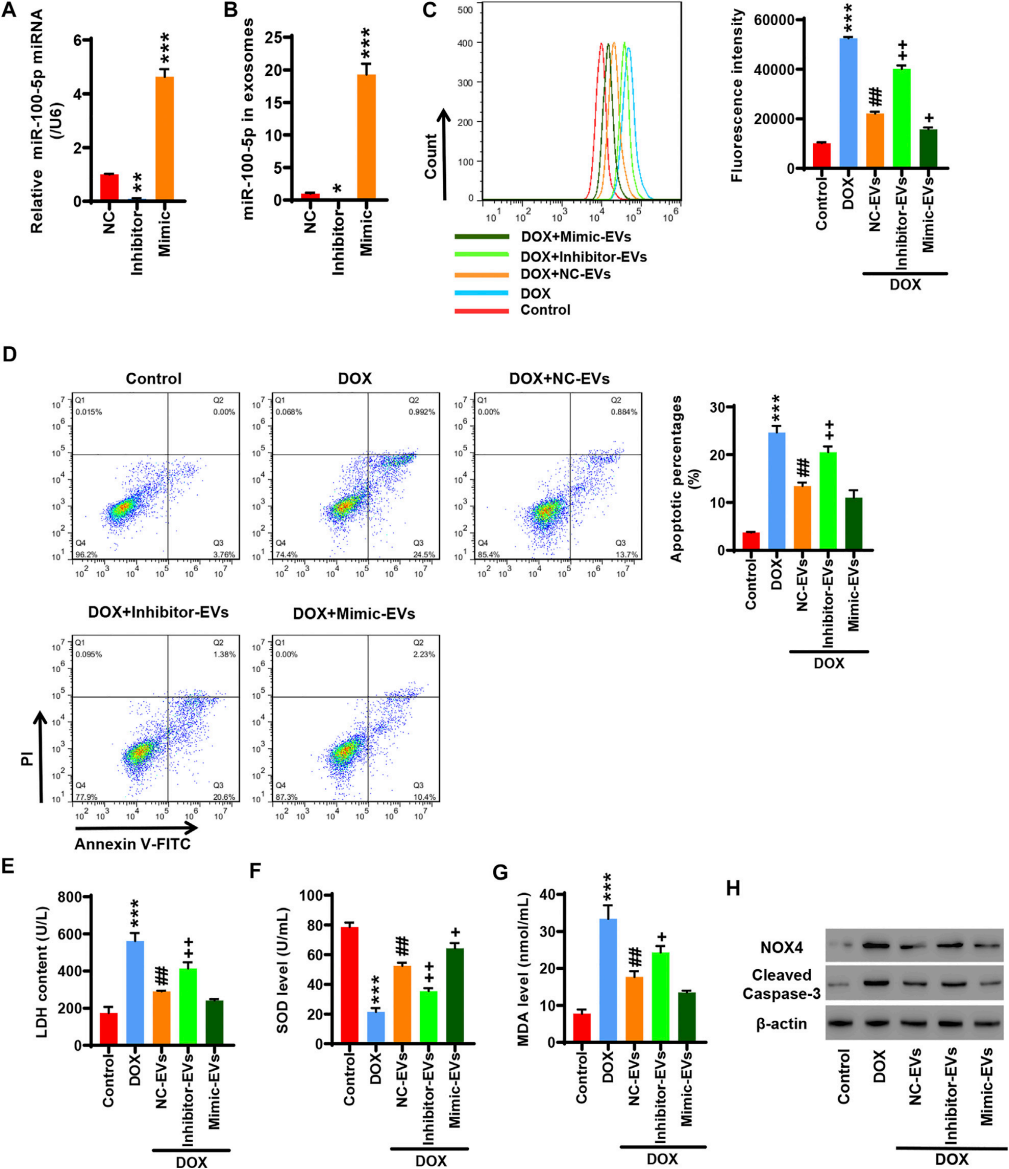
Inhibition of EV miR-100-5-p reverses the effects in which HucMSC-EVs inhibit DOX-induced oxidative stress and apoptosis. **(A)** HucMSCs were transfected with miR-100-5p inhibitor (Inhibitor) or miR-100-5p mimic (Mimic). MiR-100-5p expression was determined by Q-PCR. **(B)** HucMSCs were transfected with miR-100-5p inhibitor or miR-100-5p mimic. After HucMSC-EVs were extracted, EV miR-100-5-p was determined by Q-PCR. ^*^ *p* < 0.05, ^**^ *p* < 0.01, ^***^ *p* < 0.001 vs. NC. **(C–H)** AC16 cells were treated with 2 µmol/L DOX and EV miR-100-5p inhibitor (Inhibitor-EVs) or EV miR-100-5-p mimic (Mimic-EVs) at a concentration of 100 µg/ml. **(C)** ROS levels were measured by flow cytometry. **(D)** Apoptosis was determined by flow cytometry. Quadrant 1 represented dead cells. Quadrant 2 represented late apoptotic cells. Quadrant 3 represented early apoptotic cells, and Quadrant 4 represented normal cells. **(E–G)** LDH, SOD, and MDA levels were measured with biochemical assay. **(H)** NOX4 and cleaved-caspase-3 expression were examined by Western blot. ^***^ *p* < 0.001 vs. control, ^##^ *p* < 0.01 vs. 2 µmol/DOX, ^+^ *p* < 0.05, ^++^ *p* < 0.01 vs. 2 µmol/L DOX + NC-EVs.

HucMSCs were transfected with miR-100-5p inhibitor (Inhibitor) or miR-100-5p mimic (Mimic). Q-PCR displayed that miR-100-5p expression was abolished by miR-100-5p inhibitor, while the miR-100-5p expression was aggravated by miR-100-5p mimic (Figure 3A). After HucMSCs were transfected with miR-100-5p inhibitor or miR-100-5p mimic, HucMSC-EVs were extracted, and Q-PCR was performed. Q-PCR showed that miR-100-5p inhibitor abolished miR-100-5p expression in EVs compared to the negative control, whereas miR-100-5p mimic exceedingly exacerbated miR-100-5p expression in EVs (Figure 3B). AC16 cells were treated with 2 µmol/L DOX and 100 μg/ml miR-100-5p negative control in HucMSC-EVs (NC-EVs) or miR-100-5p inhibitor in HucMSC-EVs (Inhibitor-EVs) or miR-100-5p mimic in HucMSC-EVs (Mimic-EVs) for 24 h. Flow cytometry demonstrated that DOX remarkably increased ROS levels, which were decreased with supplement of NC-EVs or Mimic-EVs. Compared to NC-EVs, Inhibitor-EVs reversed those effects in which NC-EVs decreased ROS production which were increased by DOX, whereas Mimic-EVs had similar inhibitory effects to NC-EVs for ROS production (Figure 4C). Biochemical assay exhibited that 2 µmol/L DOX aggravated LDH release and MDA level increases as well as SOD level decreases, which were inhibited with supplement of NC-EVs or Mimic-EVs. In comparison to the NC-EV group, Inhibitor-EVs reversed those effects in which NC-EVs inhibited DOX induced the increases of LDH and MDA levels as well as the decreases of SOD levels, while Mimic-EVs had the similar effects to NC-EVs (Figures 4E–G). Furthermore, apoptosis was examined with flow cytometry and Western blot. NC-EVs and Mimic-EVs markedly reduced the percentages of apoptotic cells, which were induced to increase y DOX. Compared to the NC-EV group, Mimic-EVs had a similar effect to NC-EVs for reducing the percentages of apoptotic cells, while inhibitor-EVs reversed those effects in which NC-EVs inhibited DOX-induced apoptotic cell increases (Figure 4D). NOX4 and cleaved-caspase-3 protein expression were apparently attenuated by NC-EVs or Mimic-EVs, which was induced to increase by DOX. Compared to the NC-EV group, Inhibitor-EVs reversed those effects which NC-EVs decreased, NOX4 and cleaved-caspase-3 protein expression, which was increased by DOX, while Mimic-EVs had a similar effect to NC-EVs (Figure 4H). Taken together, these findings suggest that downregulation of EV miR-100-5-p reverses those effects in which HucMSC-EVs inhibit DOX-induced oxidative stress and apoptosis.

### Overexpression of NOX4 Cancels Those Effects in Which Human Umbilical Cord Mesenchymal Stem Cells Inhibit Doxorubicin-Induced Oxidative Stress and Apoptosis

**FIGURE 5:**
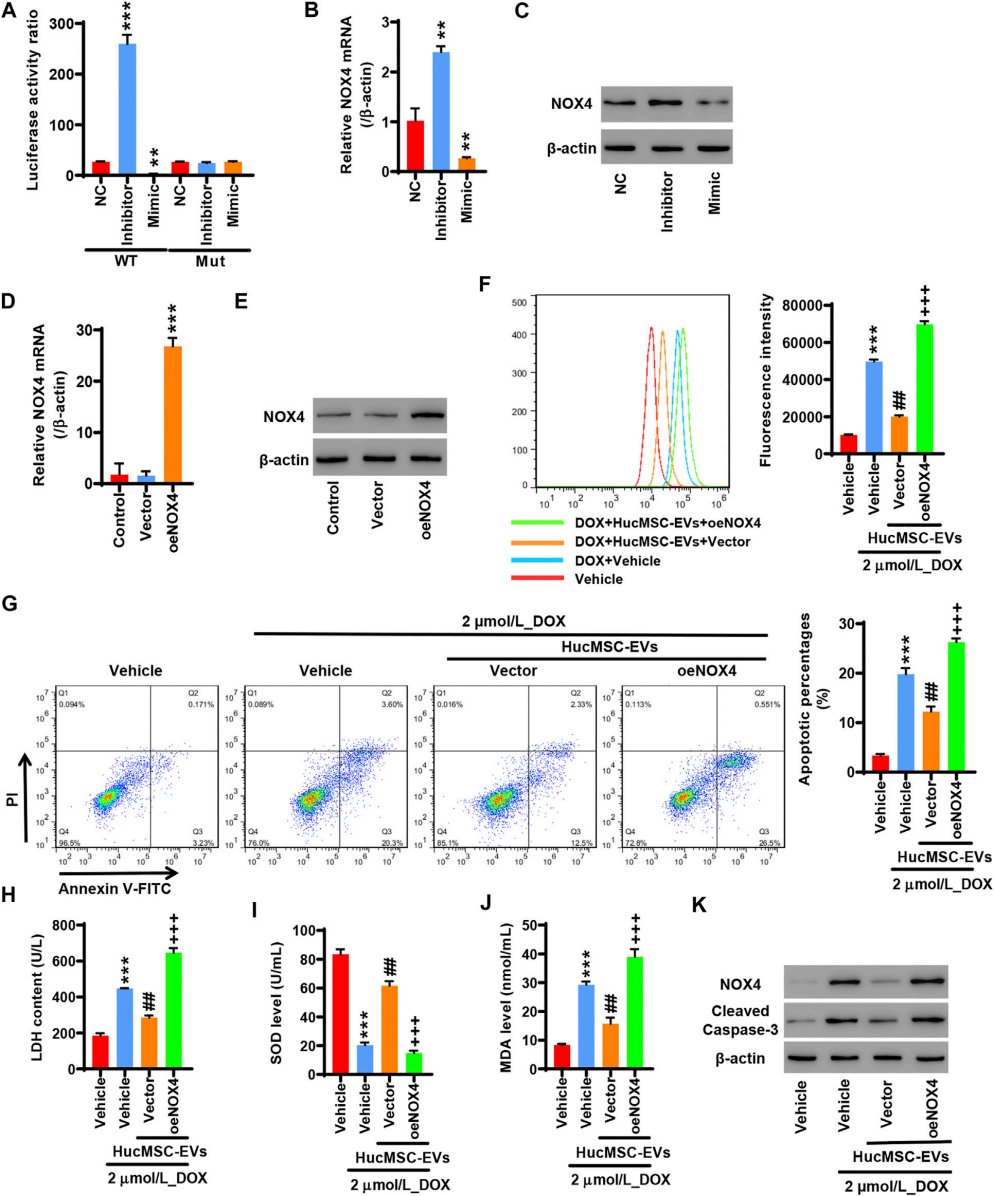
Overexpression of NOX4 abolishes those effects in which HucMSC-EVs inhibit DOX-induced oxidative stress and apoptosis. 293T cells were co-transfected with either pGL3-NOX4-WT, miR-100-5p inhibitor (Inhibitor) and miR-100-5p Mimic, and pGL3-NOX4-MUT, mimic (Mimic) or pGL3-NOX4-MUT, miR-100-5p inhibitor (Inhibitor) and miR-100-5p mimic (Mimic). **(A)** Luciferase activity was quantified using a luminometer. ^**^ *p* < 0.01, ^***^ *p* < 0.001 vs. WT + NC. **(B)** NOX4 mRNA was examined by qRT-PCR. **(C)** NOX4 protein expression was detected by Western blot. ^**^ *p* < 0.01 vs. NC. **(D)** AC16 cells were transfected with oeNOX4. NOX4 mRNA was examined by qRT-PCR. **(E)** AC16 cells were transfected with oeNOX4. NOX4 protein was detected by Western blot. ^***^ *p* < 0.001 vs. vector. **(F–K)** AC16 cells were transfected with oeNOX4 for 24 h and then treated with 2 µmol/L DOX and 100 µg/ml HucMSC-EVs for 24 h. **(F)** ROS levels were measured by flow cytometry. **(G)** Apoptosis was measured by flow cytometry. Quadrant 1 represented dead cells. Quadrant 2 represented late apoptotic cells. Quadrant 3 represented early apoptotic cells, and Quadrant 4 represented normal cells. LDH **(H)**, SOD **(I)**, and MDA **(J)** were measured by biochemical assay. **(K)** NOX4 and cleaved-caspase-3 expression were determined by Western blot. ^***^ *p* < 0.001 vs. vehicle, ^##^ *p* < 0.01 vs. 2 μmol/L_DOX + vehicle, ^+++^ *p* < 0.001 vs. 2 µmol/L_DOX + HucMSC-EVs + vector.

To study whether miR-100-5p targets the NOX4 protein, 293T cells were co-transfected with pGL3-NOX4-WT, miR-100-5p inhibitor (Inhibitor), and miR-100-5p mimic (Mimic) or pGL3-NOX4-MUT, miR-100-5p inhibitor (Inhibitor), and miR-100-5p mimic (Mimic). The miR-100-5p inhibitor markedly increased the luciferase activity of pGL3-NOX4-WT, while the miR-100-5p mimic obviously reduced the luciferase activity of pGL3-NOX4-WT. There was no change in the luciferase activity of pGL3-NOX4-MUT (Figure 5A). qRT-PCR exhibited that the miR-100-5p inhibitor increased the NOX4 mRNA expression, while the miR-100-5p mimic decreased the NOX4 mRNA expression (Figure 5B). Western blot illustrated that the NOX4 protein expression was increased by the miR-100-5p inhibitor, whereas the NOX4 protein expression was reduced by the miR-100-5p mimic (Figure 5C). These results demonstrate that NOX4 is the targeting protein of miR-100-5p. NOX4 expression is negatively regulated by miR-100-5p. Next, NOX4 overexpression plasmid (oe-NOX4) was constructed and transfected to AC16 cells. oe-NOX4 markedly increased NOX4 mRNA expression (Figure 5D) and NOX4 protein expression (Figure 5E). Furthermore, AC16 cells were transfected with oe-NOX4 for 24 h, and then treated with 2 µmol/L DOX combined with 100 μg/ml HucMSC-EVs for another 24 h. Flow cytometry revealed that HucMSC-EVs reduced ROS levels which were induced to increase by DOX, and this was reversed by the overexpression of NOX4 (Figure 5F). At the same time, HucMSC-EVs inhibited increased LDH and MDA levels as well as decreased SOD levels which were induced by DOX; these effects were reversed by the overexpression of NOX4 (Figures 5H–J). Moreover, DOX induced the percentages of apoptotic cell increase. HucMSC-EVs ameliorated the percentages of apoptotic cells which were increased by DOX, and this was reversed by NOX4 overexpression (Figure 5G). Western blot showed that HucMSC-EVs ameliorated the expression of NOX4 and cleaved-caspase-3 which was increased by DOX, and this was reversed by NOX4 overexpression (Figure 5K). Collectively, these results indicate that overexpression of NOX4 abolishes those effects in which HucMSC-EVs inhibit DOX-induced oxidative stress and apoptosis.

## Discussion

With the aging of the population, the incidence of cardiovascular diseases such as hypertension and coronary heart disease has increased significantly. The consequence of the development of most cardiovascular diseases is heart failure, which has increasingly become a major disease that seriously threatens human health. Heart failure is manifested as insufficient cardiac output and the inability to maintain the oxygen supply required by the body's metabolism. It is an important reason for the loss of labor and death in patients with cardiovascular diseases. In recent years, MSCs are of great significance for the treatment of heart failure (Vrtovec et al., 2013; Narita and Suzuki, 2015; Guijarro et al., 2016; Bartunek et al., 2017). However, MSCs have a short curative effect time, whereas MSC-derived EVs have a long curative effect time. MSC-derived EVs have a protective effect on heart failure (Chen et al., 2020b; Tan et al., 2020; Zheng et al., 2020). Chen et al. reported that EVs derived from MSCs improved cardiac hypertrophy, heart function, fibrosis, and myocardial apoptosis after transverse aortic constriction (TAC). It may be a benefit for treatment of heart failure (Chen et al., 2020a). Gao et al. found that after myocardial infarction (MI), serum EVs were obtained from ischemic heart and kidney. Cardiorenal EV-derived miRNA-1956 promoted adipose-derived MSC-mediating angiogenesis, which is important for ischemic tissue repair (Gao et al., 2020). Nakamura et al. demonstrated that injection of adiponectin and hMSC significantly increased hMSC-derived EV release. Adiponectin accelerated hMSC-derived therapy in heart failure mice (Nakamura et al., 2020). In this study, DOX induced oxidative stress, ROS, and apoptosis increases in AC16 cells, which were inhibited by HucMSC-EVs, and these were further reversed by the inhibition of EV miR-100-5-p. Meanwhile, HucMSC-EVs inhibited NOX4 expression which was induced by DOX. Overexpression of NOX4 abolished the effects of HucMSC-EVs. NOX4 was negatively regulated by miR-100-5p. These data suggest that HucMSC-EVs have protective effects against DOX-induced heart failure in AC16 cells.

Oxidative stress refers to the pathological process in which the balance of the oxidation system and antioxidant system causes an increase in ROS in the body and causes cell oxidative damage. ROS is mainly produced by the mitochondria (Dietl and Maack, 2017). ROS is also produced by the NADPH oxidase (Shang et al., 2019). Oxidative stress in the myocardium causes the increase of ROS and causes damage of the cell membrane to release LDH. MDA is the final product of lipid oxidation. SOD is the most important member of the antioxidant system. In heart failure, SOD is decreased, and LDH, MDA, and ROS are increased (Agostini et al., 2015; Casieri et al., 2017; Zhou and Tian, 2018; Koju et al., 2019; Ni et al., 2019). Cardiomyocyte oxidative stress plays an important role in heart failure (Kim et al., 2020). In heart failure, oxidative stress is markedly increased (Wang et al., 2019; Lubrano and Balzan, 2020). Inhibition of oxidative stress in cardiomyocytes can improve the symptoms of heart failure (Kumar et al., 2019; Pop et al., 2020). Heart failure is associated with oxidative stress and apoptosis. Recent studies find that apoptosis and mitochondrial oxidative stress are markedly increased in heart failure rats. Whereas myocardial capillary and arteriolar density as well as SIRT1, FOXO3a, and MnSOD expression are decreased. Echinacoside improves the heart function by the SIRT1/FOXO3a/MnSOD signaling pathway in heart failure rats (Ni et al., 2021). In DOX-induced heart failure mice, SOD2, GPx-1, FOX3a, and SIRT3 expression are decreased, apoptotic cells and cleaved-caspase-3 expression are increased, as well as inflammatory cytokines such as IL-1β, IL-6, and TNF-α are increased. LongShengZhi capsule (LSZ) is a traditional Chinese medicine. After treatment of heart failure mice with LSZ, the indicators of heart failure are significantly improved. LSZ decreases oxidative stress, apoptosis, and inflammatory cytokine levels, which are increased by DOX. LSZ increases FoxO3a, SIRT3, and SOD2 expression, which are decreased by DOX (Xu et al., 2020). In our studies, HucMSC-EVs inhibited ROS production in a concentration-dependent manner, which was induced by DOX. LDH and MDA levels were decreased by HucMSC-EVs, which were increased by DOX. SOD levels were increased by HucMSC-EVs, which were decreased by DOX. HucMSC-EV treatment was concentration dependent. Additionally, HucMSC-EV treatment decreased apoptotic cells and cleaved-caspase-3 expression in a concentration-dependent fashion, which were increased by DOX. These results suggest that HucMSC-EVs inhibit DOX-induced heart failure in a concentration-dependent manner in AC16 cells.

Nicotinamide adenine dinucleotide phosphate (NADPH) oxidase is a peroxidase. There are NOX1, NOX2, NOX3, NOX4, NOX5, DUOX1, and DUOX2 in NADPH oxidase (NOX) family (Brandes et al., 2010). NOX2 and its homologue NOX4 function as the core catalytic subunit of NADPH oxidase, which are the key to the function of the enzyme. NADPH oxidase produces ROS as a signal molecule to participate in the signal transduction process that regulates cell proliferation, senescence, and apoptosis (Ray et al., 2011; Schröder et al., 2012; Guo and Chen, 2015). When NOX family proteins are abnormally expressed, ROS levels increase, which cause oxidative stress and participate in the formation of heart failure (Kumar et al., 2019). The main function of NOX family is to generate ROS (Panday et al., 2015). Previous studies found that NOX4 knockout mice exhibited severe cardiac hypertrophy and contractile dysfunction after pressure overload, whereas NOX4 transgenic mice showed enhanced angiogenesis and increased expression of VEGF and Hif1α as well as less cardiac hypertrophy after pressure overload. It was suggested that NOX4 had a protection against cardiac stress by pressure overload (Zhang et al., 2010). Cardiac-specific overexpression of NOX4 activates the nuclear transcription factor Nrf2 (Brewer et al., 2011). Nrf2 is a key transcription factor in the cellular antioxidative stress system. Nrf2 enters the nucleus and binds to ARE (antioxidant response elements) to generate GSTs and SOD, and play a role of antioxidant damage. Nrf2 shows a protection against load-induced cardiac hypertrophy (Li et al., 2009; Schröder et al., 2012). However, recent studies demonstrate that in heart failure rats, TLR4 and NOX4 expression is significantly increased, autophagy and ferroptosis are enhanced, as well as the heart function is abnormal. Downregulation of either TLR4 or NOX4 inhibits autophagy and ferroptosis, and obviously improves cardiac function and left ventricular remodeling (Chen et al., 2019). DOX-induced cardiac and renal toxicities have been reported (Uygur et al., 2014; Tulubas et al., 2015). DOX induces apoptosis and oxidative stress in heart and kidney tissues. NOX4 is also induced to increase by DOX in the renal tissues. Pretreatment with omega-3 fatty acids improves the cardiac function, illustrates antioxidant and antiapoptotic effects, and increases renal NOX4 expression (Saleh et al., 2020). In our experiments, HucMSC-EVs ameliorated the expression of NOX2 mRNA and NOX4 mRNA, which was enhanced by DOX. Similarly, HucMSC-EVs attenuated the expression of NOX2 and NOX4 proteins, which was increased by DOX. HucMSC-EV treatment was concentration dependent. Furthermore, HucMSC-EV treatment attenuated the expression of NOX4 mRNA and protein in a time–concentration–dependent fashion, which was induced to increase by DOX. Moreover, overexpression of NOX4 abolished that HucMSC-EVs inhibited DOX-induced ROS production, increased LDH and MDA, and decreased SOD. Overexpression of NOX4 abolished that HucMSC-EVs inhibited DOX-induced increased apoptotic cells, as well as cleaved-caspase-3 and NOX4 protein expression. These data indicate that HucMSC-EVs inhibit DOX-induced heart failure through targeting NOX4 in AC16 cells.

Research in recent years has shown that miRNAs are important epigenetic regulatory factors. miRNAs also play the important roles in EVs, which are benefit to treat cardiovascular diseases. EVs play a vital role in intercellular communication and their functions depend mainly on their internal contents (Vlassov et al., 2012). EVs act as miRNA carriers that carry miRNAs to nearby or distant cells. EVs act on target cells by directly releasing miRNAs to target cells through target cell endocytosis or membrane fusion, which is considered to be an important tool for intercellular signal transduction (Montecalvo et al., 2012; Zeringer et al., 2015). EVs can deliver specific miRNAs to target cells that are as an important component of the paracrine effect of stem cells. microRNAs encapsulated in EVs are the key genetic material that promotes the repair of myocardial damage. Inhibition of miR-342-5p reduces exercise-afforded cardiac protection in myocardial ischemia/reperfusion rats. MiR-342-5p agomir increases the miR-342-5p levels and decreases myocardial infarct size in rat hearts. Exercise-derived circulating EVs mediate the protective effects against myocardial ischemia/reperfusion injury through EV miR-342-5p (Hou et al., 2019). MiR-100-5p is believed to have anti-atherosclerotic effects because it inhibits the proliferation of endothelial cells and migration of blood vessels and smooth muscle cells (Shoeibi, 2020). In a mouse model of atherosclerosis, the expression of miR-100-5p can improve endothelial function, weaken atherosclerosis, and reduce plaque area (Linna-Kuosmanen et al., 2020). Further studies indicate that downregulation of miR-100-5p activates the VEGFA/MYC pathway, which leads to increased endothelial cell metabolism, proliferation, and angiogenesis, thereby promoting angiogenesis (Pankratz et al., 2018). Downregulation of miR-100 leads to the formation of angiogenic tubes, the increase in endothelial germination, and proliferation in HUVECs (Grundmann et al., 2011). NOX4 is the target of miR-100-5p. Inhibition of miR-100-5p targeting NOX4 leads to H_2_O_2_ release (Kriegel et al., 2015). The expression of miR-100-5p is decreased by hypoxia. EVs derived a human neural stem cell line that inhibits hypoxia-induced proliferation and migration through EV miR-100-5-p in pulmonary artery smooth muscle cells (Wang et al., 2020). Hromadnikova et al. found that downregulation of miR-100-5p was associated with gestational hypertension and preeclampsia (Hromadnikova et al., 2016). Onrat et al. compared 50 patients with dilated cardiomyopathy and 10 healthy persons. They found that miR-100-5p was overexpressed in the dilated cardiomyopathy (Onrat et al., 2018). In this study, HucMSC-EVs inhibited DOX-induced ROS, LDH, and MDA increases, and SOD decrease, which were reversed by the EV miR-100-5-p inhibitor. There were differences in reducing ROS levels and increasing SOD levels between EV miR-100-5p mimic treatment and HucMSC-EV treatment. But there were no differences in reducing LDH and MDA levels between EV miR-100-5-p mimic treatment and HucMSC-EV treatment. It means that the effects of EV miR-100-5p treatment and HucMSC-EV treatment are similar in reducing ROS and oxidative stress. Furthermore, HucMSC-EVs inhibited DOX-induced apoptotic cell increase, and cleaved-caspase-3 and NOX4 protein expression increase, which were reversed by the EV miR-100-5-p inhibitor, whereas the effects of EV miR-100-5p treatment and HucMSC-EV treatment were similar in reducing apoptosis. Moreover, miR-100-5p inhibited NOX4 mRNA and protein expression, whereas the inhibition of miR-100-5p increased NOX4 mRNA and protein expression. Taken together, these data indicate that EV miR-100-5-p treatment inhibits DOX-induced heart failure *via* targeting the NOX4 protein in AC16 cells, which is similar to the effects of HucMSC-EV treatment.

## Conclusion

In summary, HucMSC-EV treatment inhibited DOX-induced oxidative stress, ROS, and apoptosis increases. HucMSC-EV treatment also inhibited DOX-induced NOX4 expression. Overexpression of NOX4 abolished those effects in which HucMSC-EVs inhibited DOX-induced oxidative stress, apoptosis, and ROS production. NOX4 protein expression was negatively regulated by EV miR-100-5-p. Inhibition of EV miR-100-5-p reversed those effects in which HucMSC-EVs inhibited DOX-induced oxidative stress, apoptosis, and ROS production, whereas EV miR-100-5-p played a role similar to HucMSC-EVs in reducing oxidative stress, apoptosis, and ROS production. It is suggested that HucMSC-EVs inhibit DOX-induced heart failure through the miR-100-5p/NOX4 pathway.

## Data Availability

The datasets presented in this study can be found in online repositories. The names of the repository/repositories and accession number(s) can be found in the article/Supplementary Material.
